# Policy implementation and outcome evaluation: establishing a framework and expanding capacity for advocacy organizations to assess the impact of their work in public policy

**DOI:** 10.1186/s12961-024-01110-0

**Published:** 2024-02-20

**Authors:** Laurie P. Whitsel, Sally Honeycutt, Reyna Radcliffe, Janay Johnson, Paul J. Chase, Philip Noyes

**Affiliations:** American Heart Association, 1150 Connecticut Avenue – Suite 300, Washington, DC 20036 USA

## Abstract

Advocacy organizations can play a crucial role in evaluating whether legislation or regulation has had its intended effect by supporting robust public policy implementation and outcome evaluation. The American Heart Association, working with expert advisors, has developed a framework for effective evaluation that can be used by advocacy organizations, in partnership with researchers, public health agencies, funders, and policy makers to assess the health and equity impact of legislation and regulation over time. Advocacy organizations can use parts of this framework to evaluate the impact of policies relevant to their own advocacy and public policy efforts and inform policy development and guide their organizational resource allocation. Ultimately, working in partnership, advocacy organizations can help bring capacity, commitment and funding to this important implementation and outcome evaluation work that informs impactful public policy for equitable population health and well-being.

## Introduction

Policy implementation and outcome evaluation, which assesses whether a particular public policy has had its intended impact when implemented, is an integral component of the policy process. It provides an overall assessment of the policy effects and can guide responsible decision making for ongoing policy development. Effective evaluation of how policies are implemented, scaled and funded can also inform decision making and prioritization by policy makers, funders, advocacy organizations and public health agencies [1]. Yet, it is significantly underutilized [2–4].

Many advocacy organizations, including the American Heart Association, are interested in an evaluation framework that helps assess the impact of the public policies they work to pass and implement. A framework that examines whether their resources and investments are optimally focussed on the most effective policies that can impact population health and whether there is equity impact would help guide organizational decision making. Evaluating the impact and outcome of public policy implementation allows advocacy organizations to engage with the research community to bring additional capacity for the kind of evaluation that can lead to evidence-based, equity-focussed policy making.

Advocacy organizations support social, public health or other causes that require changes in government, public policy or systems, often related to their mission. Advocacy organizations vary significantly in size, causes, structure/function and funding. The American Heart Association has a mission of being a relentless force for a world of longer, healthier lives and has done advocacy work for more than 40 years across issues related to prevention (tobacco, nutrition security, physical activity), acute systems of care, access to care, heart and brain research, digital health, public health infrastructure and appropriations. The organization has previously described how it is structured and functions in doing its advocacy work [5, 6].

### Evidence for impact—public policy implementation and outcome evaluation

Fundamentally, for advocacy organizations to optimize their work in public policy, they need to understand whether the policies they work so hard to get into place are implemented as intended. This includes whether the policies are associated with specific population impacts, whether they increase equity or disparities, what they cost to implementers and priority populations, the degree and scale of their penetration and uptake, whether they are associated with unintended consequences, and whether they contribute to creating longer, healthier lives. Effective evaluation assesses policy adoption, acceptability, penetration, feasibility, fidelity, implementation cost, cost-effectiveness, unintended consequences and sustainability [7, 8].

Table 1 provides definitions for each of these aspects of evaluation with equity considerations. A need exists to include quantitative metrics from relevant data monitoring systems to assess objective change or progress in health and equity over time [9]. Quantitative analysis has historically been under-utilized in policy implementation and outcome evaluation and is necessary to objectively assess population impact. [9] Relevant surveillance systems must be matched with the objective measures that will be tracked over time and the lag between data collection and public reporting of the surveillance data must be taken into account. Table 2 provides an example of a cascade approach to assessing health and equity impact longitudinally with quantitative measures. Equity domains may include income, educational attainment, race/ethnicity, geography, rurality, sexual orientation, gender identity, physical and mental disability, and other considerations specific to the issue area. While these metrics will primarily demonstrate association rather than causality with the policy change, the analysis will be important for assessing any impact on population health over time.

**Table 1:** Areas of policy implementation and outcome evaluation assessment [11, 19–21]

Areas of assessment	Definition	Potential research questions	Equity considerations
Adoption	The number, percentage and representativeness of jurisdictions that pass or enact the policy, including allocation of resources for implementation and enforcement	What is the policy mechanism employed (e.g., executive order, legislation, regulation, contracts)? At what level of government was the policy addressed? Is the policy based on evidence-based scientific or clinical recommendations? Was the policy formally approved or passed? Was the policy adopted? If so, what was the adoption date? What is the scope or elements of the policy and at what level (i.e. federal, state, local) is it being adopted/implemented. Were implementation process steps outlined in statute and regulation? If so, capture language. Was implementation evaluation written into the law? Was the policy implemented? If so, was there an implementation date? What resources and funding were available to ensure implementation of the policy? Are resources or training available to support lower capacity implementors to ensure equity? What existing social, political or economic realities were occurring at the time of implementation of this policy?	Is there equity in where the policy was passed? Is there equity in where it was implemented? Is there equity in the funding resources? Is there equity in the training resources? Is there over-enforcement or under-enforcement in some locations or with some community groups?
Penetration/reach	The number, percentage and representativeness of individuals affected by the policy and the systems changed or processes improved with policy implementation	Scope/reach of the policy (actual population reach, systems change, process improvement). Does the policy address any social determinants of health? Was there a system change with this policy? What was the actual population reach for this policy (especially noting across race/ethnicity, geography, income, education? Did this policy improve or worsen any existing processes?	Is there consideration for how the policies reach those at the greatest risk or in the greatest need? Is there equity in the reach and penetration for the benefits to all locations and all community groups? Is there equity in the burdens to all locations and all community groups?
Acceptability	The perception among relevant audiences, including decision makers, implementers and intended beneficiaries, that the policy is agreeable, acceptable or adequate	Who were the collaborators involved in adoption and implementation? Were people who would be most impacted by the policy involved in implementing? What supporting policies, resources (e.g., technical assistance, training) and/or processes were put in place to enforce the implementation of the policy? Was the policy well received across all priority populations? If so, by whom? If not, by whom? Was the policy change adequately communicated? If so, by whom/by what agency or stakeholder? How did the communication happen? What was the level of engagement with enactment? Amongst implementers, how was the policy received across impacted populations? Did the population of focus know about the policy and support it?	Assure that those in the community can provide feedback and co-create policy solutions and prioritization. Who was consulted in the development of the policy approach? Who decides the acceptability of the policy approach? Is the policy approach culturally relevant and affirming to all groups? Is the policy approach a priority to all groups? Is feedback from all locations and all community groups considered? Which locations and community groups were engaged in how the policy would be implemented? How is the policy approach communicated and who are the spokespeople?
Feasibility	The extent to which the policy can be successfully implemented in a particular community or setting	Were implementers adequately trained to implement all aspects of the policy? Were there barriers to implementation? If so, what were they? Were they overcome? If so, how? What/who were the facilitators for implementation?	Is the policy approach feasible for all locations and all community groups? Are there resources to address unequal barriers and burdens?
Fidelity	The extent to which the policy was implemented as intended	Was the policy implemented as intended?	Were differences by locations and community groups considered so that the policy could be consistently implemented as intended (i.e. socio-cultural, geographic and other areas of diversity)
Implementation cost	The value of all resources required to implement the policy, including time, facilities and materials	What was the cost of implementation? Are there any annual appropriations in place associated with implementation?	Was the unequal burden of costs considered for locations and community groups that are under-invested? For locations and community groups that are under-invested, will the implementation costs take resources from other areas?
Effectiveness	Change in intended outcomes, appropriate to the amount of time since the policy was adopted. Successful policy implementation may require years or even decades to change health outcomes; changes in environments or behaviours may be appropriate short-term outcomes	Was the policy implemented as intended? Did it have its expected outcomes? Was it implemented equitably across different communities, populations or geographies?	Is the policy approach effective for all groups and all community groups? Will the policy approach close absolute and relative gaps between groups?
Unintended consequences	Any outcomes (positive or negative) that were not intended by those who developed or adopted the policy. [21]	Were there any unintended consequences experienced by the priority population or other population groups? If so, describe. Were there any unintended consequences experienced during implementation at the systems level? If so, please describe. Were disparities increased or decreased with implementation?	Are there disproportionate unanticipated consequences for under-invested locations and community groups? Are unintended consequences equitably addressed for all groups?
Sustainability	Maintenance of the policy over time. Includes continued implementation, enforcement, and resulting outcomes. [19]	Was the policy change sustained over time? How long? Did outcomes vary over time? If so, how? Was the implementation process sustainable over time?	Maintenance of the policy over time. Includes continued implementation, enforcement and resulting outcomes. Assessment should focus on two levels: communities reached by the policy, and jurisdictions that enact the policy. [19] Are there differences for locations and community groups in how policy approaches are maintained over time?
Monitoring	Assessing the impact of the law through surveys and other data monitoring systems	Is there capacity for monitoring? If so, capture the language (roles and responsibilities; who is responsible for monitoring?). Is there a role for a federal, state and or local health agency and any dedicated appropriations/funding for evaluation?	Are there differences in the burden or risks of monitoring for some locations and community groups?

**Table 2:** Examples of longitudinal approach to quantitative health and equity assessment in policy implementation and outcome evaluation

Example issue	First outcome measure (monitoring)	Second outcome measure (monitoring)	Third outcome measure (monitoring)	Fourth outcome measure (monitoring)
***Tobacco***				
Excise taxes	Tobacco price increase (TFK tracking)	Youth tobacco use prevalence (NYTS, YRBSS, Monitoring the Future)	Adult tobacco use prevalence (BRFSS, PATH)	MI rates and other CVD outcomes (Vital Statistics)
Comprehensive clean indoor air	Population no./% covered by the law/ordinance (AHA tracking)	Youth tobacco use prevalence, second-hand smoke exposure (NYTS, YRBSS, Monitoring the Future)	Adult tobacco use prevalence, second-hand smoke exposure (BRFSS)	MI rates and other CVD outcomes (Vital Statistics)
*Access to care*				
Medicaid expansion	No. of states expanding and implementing (AHA tracking)	No. of new enrollees covered (AHA tracking)	No. of people with access to preventive services	CVD outcomes (MI, hypertension, diabetes) Vital Statistics
Access to coverage	No. of people covered	No. of people with access to preventive services	CVD outcomes (MI, hypertension, diabetes) Vital Statistics	
Physical activity				
Complete streets	Policy implemented	Infrastructure improvements	Population reach	Self-reported measures of PA (BRFSS, YRBSS)
Early care and education licensing regulations (including PA standards)	No. of facilities (centre/home-based) that fully met the standards (e.g., CDC/NRC)	No. of children covered (facility/home-based care)	Licensing official trainings that occurred; embedded in compliance visits	Young children’s physical activity levels (CDC’s Childcare Survey of Activity and Wellness)
Healthy diet				
Sugary beverage taxes	Increase in cost of beverages (likely will have to purchase these data)	Beverage sales and changes across the beverage portfolio	Reported beverage consumption in youth and adults (YRBSS, BRFSS)	CVD outcomes, obesity and diabetes rates (Vital Statistics, LHANES/NHANES, BRFSS, YRBSS)
SNAP	Level of appropriations	Fruit and vegetable purchases	Self-reported fruit and vegetable intake (YRBSS, BRFSS, LHANES/NHANES)	

*AHA* American Heart Association, *BRFSS* Behavioral Risk Factor Surveillance System, *CDC* Centers for Disease Control and Prevention, *CVD* cardiovascular disease, *LHANES* Local Health and Nutrition Examination Survey, *MI* myocardial infarction, *NHANES* National Health and Nutrition Examination Survey, *NRC* National Resource Center, *NYTS* National Youth Tobacco Survey, *PA* physical activity, *PATH* Population Assessment of Tobacco and Health, *TFK* Tobacco-Free Kids, *YRBSS* Youth Risk Behavior Surveillance System

### The framework

**Fig. 1:**
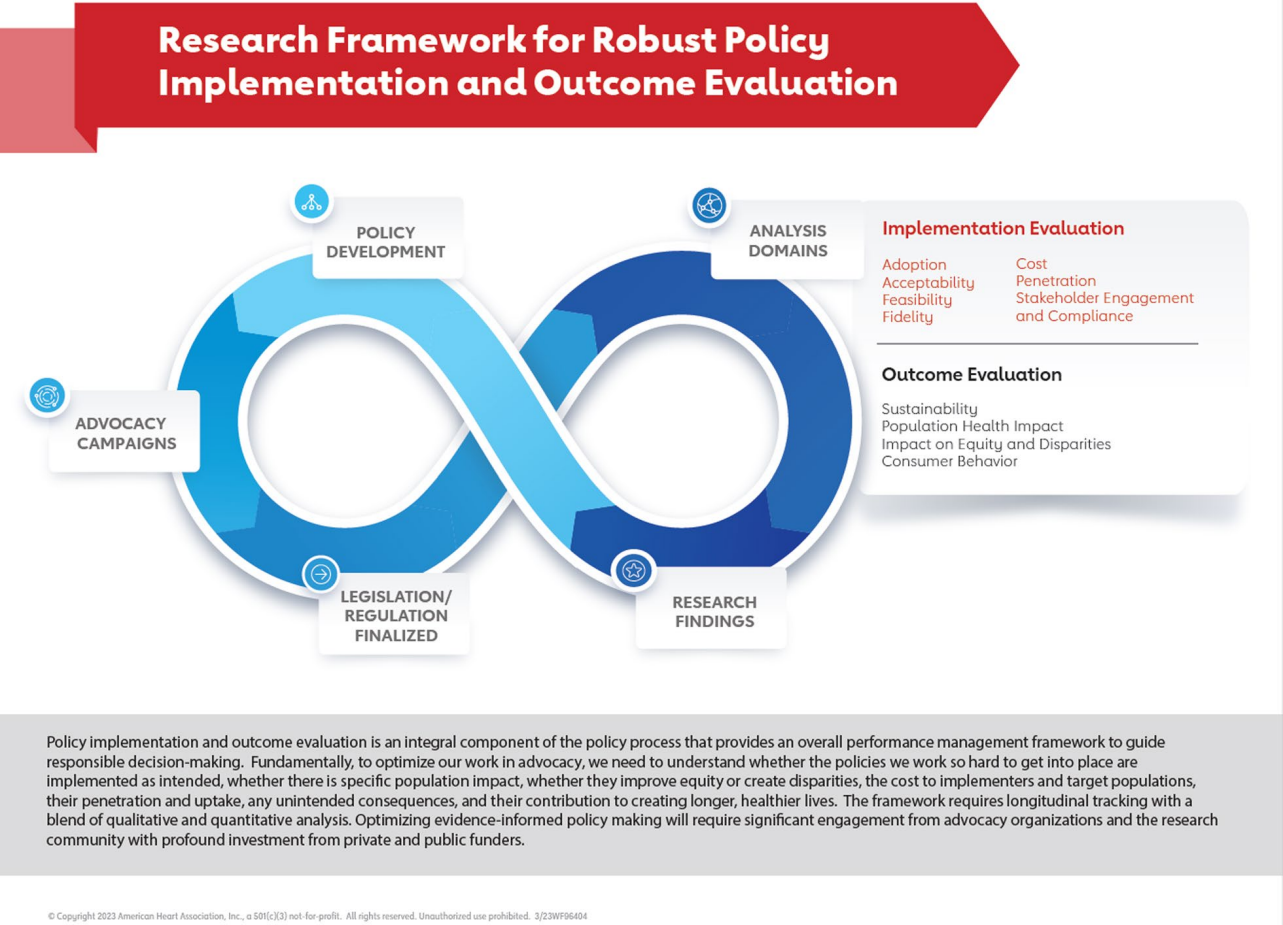
Research Framework

Although there are a range of existing frameworks, [10–12], most focussed on program implementation. There is a need for a more robust public policy implementation and outcome evaluation framework that can be used by non-profit advocacy organizations, researchers, government agencies and other key collaborators. In 2019, the American Heart Association’s policy research team convened an expert advisory group to help develop such a framework. The policy research team translates science and evidence base into impactful public policy in the areas of cardiovascular disease and stroke prevention and health promotion. The association’s policy research team provides policy development and the foundation for AHA’s advocacy work at the global, national, state and local levels. The expert advisory group it convened was comprised of individuals representing the funding community that has invested in community-led programmes and leading researchers and clinicians. Each of these individuals are recognized in the acknowledgements. The group met for about a year with the objective of informing a draft framework that was completed at the end of the convenings. The policy research team gathered further internal and external feedback, including from key staff at the Centers for Disease Control and Prevention, and finalized the framework. The Fig. 1 provides a visual representation of the conceptual framework that can be applied across public policy issues. This evaluation framework calls for longitudinal assessment and blends qualitative and quantitative analysis.

As we move forward into implementing parts of the framework, we will convene additional expert advisory groups to inform our evaluation and we will purposefully include people with lived experience.

### Equity considerations

Implementation and outcome evaluation is essential to optimize public policy efforts that aim to address inequities. Long-standing health disparities stemming from the historical, inequitable distribution of wealth, power and privilege and exist across almost every health indicator and outcome [13]. There is a need for equitable policy, systems and environment changes that are rooted in an understanding of the historical arc of structural racism and the influence of structural inequities on the proliferation of health-compromising conditions and the American Heart Association has issued a call to action identifying structural racism as a fundamental driver of health disparities [14]. Advocacy organizations can help catalyse public policy making at all levels of government to cultivate environments that support population health and well-being [13]. Policies are frequently passed with exaggerated claims of reducing inequities. Without implementation and outcome evaluation, there is little or no accountability measure to determine if those claims are met. Although there may be more attention and discussion of heath equity recently, research shows that this has not necessarily been translated into increased equity as policies are implemented. The overall health equity in mortality, for example, has slowed in recent decades, not accelerated [15].

The importance of evaluation and accountability is highlighted in *What is Health Equity* brief developed by the Robert Wood Johnson Foundation [16]. It outlined three essential elements to achieve health equity as follows:

- The approach addresses the underlying, cross-sector social inequities that are needed to be healthy.
- The approach is focussed to produce the greatest benefit for groups that live in or are members of disadvantaged or under-invested communities.
- The impact is evaluated, not by measuring the average impact on the whole population, but by examining the impact in under-invested communities, as well as comparing the absolute and relative differences with advantaged communities.

Engagement of under-invested communities is needed from the beginning of policy advocacy work. At each stage of the policy implementation and outcome evaluation process there are important questions to examine the potential impact on locations and community groups most burdened by inequities.

The review by Brownson et al. provides the following 10 recommendations as a pathway for advancing health equity through implementation science: ‘(1) link social determinants with health outcomes, (2) build equity into all policies, (3) use equity-relevant metrics, (4) study what is already happening, (5) integrate equity into implementation models, (6) design and tailor implementation strategies, (7) connect to systems and sectors outside of health, (8) engage organizations in internal and external equity efforts, (9) build capacity for equity in implementation science, and (10) focus on equity in dissemination efforts’[17].

### Partnership engagement and collaboration

Advocacy organizations cannot do policy implementation and outcome evaluation unilaterally. They must do it in partnership with academia, public health departments, funders, coalition partners, those with lived experience and other key collaborators. Only through partnering and collaboration can organizations bring the necessary capacity to this work. There are several key steps that advocacy organizations can take to facilitate greater commitment and collaboration around public policy evaluation. Depending on their capacity, mission-focused priorities, partner engagement, and community context, advocacy organizations may focus on some or all of these key steps:

- Make it a standard of practice to advocate for and ensure appropriations for the monitoring and evaluation of a law or regulation.
- Convene the research community and key collaborators, including public health departments, to develop an evergreen research agenda. Bring feedback from field and federal advocacy to the research community and funders to help support policy-relevant research.
- Develop relationships with career professionals in the regulatory agencies across all levels of government to help support and monitor policy implementation.
- Commit to some level of evaluation at the organizational level to assess the impact of public policies the organization has worked to pass.
- Conduct and/or secure funding for message testing research funded to better make the case to policy makers for the importance of policy implementation and outcome evaluation and why it needs to be appropriated.
- Support public/private resources and partnerships to support technical assistance in implementation and engage lived experience into policy outcome evaluation.
- Enable, through partnerships with citizen groups/organizations, the ability to study public policy implementation and outcomes in real time and monitor ongoing refinement [18].

## Conclusion

If advocacy organizations can commit to facilitating, initiating and advocating for policy implementation and outcome evaluation in public policy, there is the opportunity to develop more impactful policy, understand the health and equity impact of legislation and regulation across the population, and guide organizational decision making and resource commitment. Engagement with funders, key collaborators, the research community, policy makers, government agencies, those with lived experience and public health is essential to bring the necessary capacity, resources and commitment to this work. Partnering with public health agencies is especially important to optimize the impact of funding to states and communities, improve population health monitoring and inform public health frameworks. The American Heart Association will use the framework to guide efforts to better capture the reach and impact of the public policies that we have worked to pass. Capturing the reach and impact of enacted policies will help us understand how our advocacy efforts have contributed to our organizational impact goal and strategic priorities. We will also disseminate this framework to our key partners and collaborators and to the research community. Together, with significant collaboration and coordination to achieve robust public policy implementation and outcome evaluation, the American Heart Association and other advocacy organizations can play an important role in informing the most effective public policy strategies to support population health and well-being.

## Data Availability

Not applicable.
